# Can a 5-to-90-day Mortality Predictor Perform Consistently Across Time and Equitably Across Populations?

**DOI:** 10.1007/s10916-023-01962-z

**Published:** 2023-07-03

**Authors:** Jonathan Handler, Olivia J. Lee, Sheena Chatrath, Jeremy McGarvey, Tyler Fitch, Divya Jose, John Vozenilek

**Affiliations:** 1 Clinical Intelligence and Advanced Data Lab, OSF Healthcare System, 1306 N Berkeley Ave, Peoria, IL 61603 USA; 2 University of Illinois College of Medicine at Peoria, Peoria, IL USA; 3 Ministry Healthcare Analytics, OSF HealthCare System, Peoria, IL USA; 4 Internal Medicine and Pediatrics, OSF Healthcare System, Peoria, IL USA; 5 Business Intelligence Consulting, Indus Group, Wheeling, IL USA; 6 Department of Emergency Medicine, Northwestern University Feinberg School of Medicine, Chicago, IL USA; 7 OSF Innovation, OSF Healthcare System, Peoria, IL USA; 8 University of Illinois College of Engineering, Urbana Champaign, Champaign, IL USA

**Keywords:** Mortality, Prognosis, Machine learning, Health equity, Advance care planning, COVID-19

## Abstract

Advance care planning (ACP) facilitates end-of-life care, yet many die without it. Timely and accurate mortality prediction may encourage ACP. However, performance of predictors typically differs among sub-populations (e.g., rural vs. urban) and worsens over time (“concept drift”). Therefore, we assessed performance equity and consistency for a novel 5-to-90-day mortality predictor across various demographies, geographies, and timeframes (n = 76,812 total encounters). Predictions were made for the first day of included adult inpatient admissions on a retrospective dataset. AUC-PR remained at 29% both pre-COVID (throughout 2018) and during COVID (8 months in 2021). Pre-COVID-19 recall and precision were 58% and 25% respectively at the 12.5% certainty cutoff, and 12% and 44% at the 37.5% cutoff. During COVID-19, recall and precision were 59% and 26% at the 12.5% cutoff, and 11% and 43% at the 37.5% cutoff. Pre-COVID, compared to the overall population, recall was lower at the 12.5% cutoff in the White, non-Hispanic subgroup and at both cutoffs in the rural subgroup. During COVID-19, precision at the 12.5% cutoff was lower than that of the overall population for the non-White and non-White female subgroups. No other significant differences were seen between subgroups and the corresponding overall population. Overall performance during COVID was unchanged from pre-pandemic performance. Although some comparisons (especially precision at the 37.5% cutoff) were underpowered, precision at the 12.5% cutoff was equitable across most demographies, regardless of the pandemic. Mortality prediction to prioritize ACP conversations can be provided consistently and equitably across many studied timeframes and sub-populations.

**Supplementary Information:**

The online version contains supplementary material available at 10.1007/s10916-023-01962-z.

## Introduction

Advance care planning (ACP, which may also refer to the resulting advance care plan) is a process to discuss and document patients’ preferences for end-of-life care [1]. Patients and clinicians agree that ACP enables each patient to receive their desired life-extending care while avoiding the pain, discomfort, social separation, and cost of end-of-life procedures that the patient does not want [2, 3]. Demonstrated ACP benefits include respecting end-of-life wishes, decreasing the burden on loved ones, stress reduction, improved patient satisfaction, and fewer in-hospital deaths [3].

Although experts agree on the importance of ACPs, clinicians cite time constraints and poor communication with other providers as barriers to having end-of-life discussions [3, 4]. Reduced access to healthcare in mixed-rurality populations may make ACP even more unlikely [5]. Due to these barriers, many patients do not have documented preferences at the end-of-life and therefore do not achieve what has been termed an “ideal death” [6–8].

Some algorithms predict mortality too early to create urgency or too late for meaningful ACP discussion. For example, the Charlson comorbidity index predicts mortality within the next ten years and may not create a sense of urgency [9], while the APACHE II and IV scores predict mortality risk for ICU patients during the current inpatient stay [10] when the ability to have meaningful discussion may be compromised (e.g., due to obtundation or mechanical ventilation) [11, 12].

Accordingly, NYU Langone Health developed an algorithm to predict mortality within 60 days after the start of an inpatient admission using data from their three medical centers in New York City. Their aim was to support identification of palliative care candidates. Their model utilized 9614 features and achieved 0.28 area under the precision-recall curve (AUC-PR) [13].

We sought a model to predict post-inpatient mortality to meet a different need – to help prioritize and encourage timely ACP conversations during an inpatient stay. Although our system aims for ACPs with every patient, time constraints and other factors can make this infeasible. Our system serves a mixed-rurality population, and rurality constraints (e.g., gaps in palliative care availability and longer travel distances for care) may further reduce ACP feasibility [7]. Predicting mortality using clinician gestalt alone may have limited accuracy, but combining gestalt with a predictive model may be synergistic [14]. Therefore, to help prioritize ACPs when resources are limited, and to encourage clinicians to have ACPs in those more likely to benefit, we developed a model to predict mortality occurring 5-to-90 days after the start of an inpatient admission. For more information about the model, see Supplement.

The model’s intended use is to predict mortality soon after the length of an average inpatient stay. Therefore, the 5-to-90 day window was chosen to: 1) begin after the average 4-day length of an inpatient stay [15], 2) allow at least 4 days for an ACP if the inpatient stay is longer than average, and 3) create enough urgency to stimulate the ACP. Since much of the data feeding the model may come from outpatient care prior to the admission, and most of the prediction window covers a period when most patients will have been discharged after the admission, the effects of home geography on mortality and its prediction, access to care, and likelihood of an ACP are highly relevant. Initial efforts to build a predictor inspired by many of the Langone model’s strongest reported features did not lead to adequate performance, so a new model had to be created for our mixed rurality population. The model appears to be novel because it was trained on a mixed-rurality population, utilizes a 5-to-90-day prediction window, and requires only 13 input features (easing implementation and the ability to explain predictions – see Table 1).

**Table 1:** Available and selected features included in the model

**Categories of Features Used for Model Development**	**Final Features Selected by the Model**
• Demographics o Gender o Race o Ethnic group o Age at the date of the encounter o National and state Area Deprivation Index (ADI) o Information about the visit • Summary information about prior visit counts • Active problem list content such as total number of problems, number of cancers • Counts of active medications on the medication list • Counts of classes of prior lab results and changes in those counts over time • Aggregate values (average, minimum, and maximum) for specific lab results and changes in those values over time • Charges for services, excluding charges from the 30 days immediately prior to the admission (as they may not yet have posted at the time of admission) • Counts of surgeries and procedures on a patient over time, changes in those counts over time • Features that apply mathematical functions to medication counts and problem counts as of the time of a visit	• Age • Minimum albumin from the time of the encounter to one month prior • Count of cancers on the patient’s active problem list • Average BNP from 12 months to 1 month prior to the encounter • Average blood albumin from time of the encounter to one month prior • Change in the minimum bilirubin comparing the period of 12 months prior to the encounter to 1 month prior to the encounter to the period of 1 month prior to the encounter to the time of the encounter • Range of BNP from the time of the encounter to one month prior • Change in the count of abnormal labs per day comparing the period of 12 months prior to the encounter to 1 month prior to the encounter to the period of 1 month prior to the encounter to the time of the encounter • Count per day of outpatient visits per day from 12 months to 1 month prior to the encounter • Average red blood cell count (RBC) from the time of the encounter to one month prior • Range of total bilirubin from the time of the encounter to one month prior • Count per day of inpatient visits per day from 12 months to 1 month prior to the encounter • Count per day of emergency department (ED) visits per day from 12 months to 1 month prior to the encounter

Algorithms can experience performance degradation over time due to “concept drift,” [16] and may perform differently across demographic groups [17]. This can lead to mistrust of the model and loss of its benefits, while varying performance across demographic groups can lead to healthcare inequities [18]. Therefore, this study assesses whether the model retains predictive performance over time (especially during a global pandemic) and performs equitably across patient subgroups.

### Objective

We sought to retrospectively assess the model’s performance over different timeframes and demographic subgroups to assess and compare its consistency and equity of performance in those contexts.

## Materials and methods

### Declarations

This study was approved with exemption determination by the University of Illinois College of Medicine at Peoria Institutional Review Board.

### Model assessment

We assessed the model on datasets retrospectively extracted from the health system’s enterprise data warehouse (EDW), which contains data from a variety of sources, including the health system’s electronic health record and another source [19] of death records. The pre-COVID dataset included visits throughout 2018 and the during-COVID dataset included visits during 8 months of 2021. Datasets contained one row per inpatient visit during the selected timeframe, including visits for patients >= 18 years of age at the time of admission, and whose resuscitation status at the time the model was assessed (a proxy for status on admission) was either “Full Code” or null. Since multiple health systems service the geography and different patients have different data elements available, we also required at least one lab test available in the EDW in the 31–365 days prior to the visit for its inclusion (to ensure at least minimal data available on which to predict). Since the model automatically adjusts for, and makes a “best effort” prediction in the face of missing data (described in the Supplement), predictions were made on every included patient. No visit used to originally develop or assess the model was used in this analysis. Although the model uses significantly engineered input features, all features are generated from a single query against the database.

Model performance was assessed by populating datasets with the input features and target variable (5-to-90-day mortality), generating a prediction using the features, and assessing performance in different timeframes, for different patient subsets, and at different certainty cutoffs. Boolean predictors produce a certainty value between 0 and 100%. Implementation teams select a certainty cutoff value to divide “yes” from “no” predictions, seeking the best tradeoff between false positives and false negatives given the intended use. To assess performance, we calculated precision (positive predictive value) and recall (sensitivity) at certainty cutoffs of 12.5% (for greater recall) and 37.5% (for greater precision), area under the receiver-operator characteristic curve (AUC-ROC), and AUC-PR. Those cutoffs were chosen by clinicians as having appropriate false positive vs. false negative tradeoffs for our intended use (based on the model’s prior performance on the development test set). All datasets ended at least 6 months prior to analysis to ensure at least 90 days had passed after the visit to populate the target variable plus another 90 days to account for death reporting delays.

**Fig. 1:**
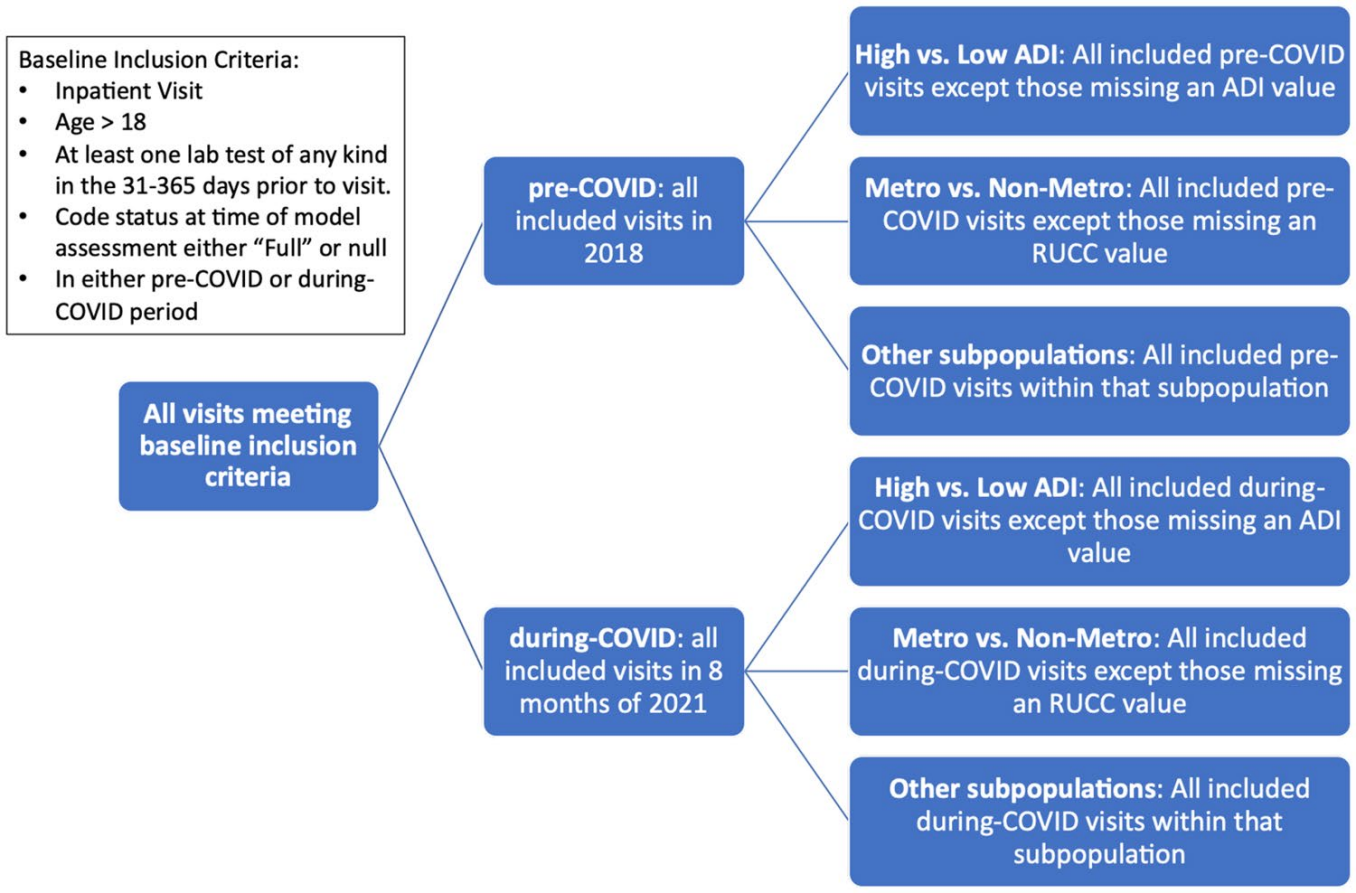
Study Inclusion/Exclusion Criteria

Performance was assessed on various demographic subgroups. Since White non-Hispanic patients represent a majority of the studied population, other race/ethnicity subgroups were combined to reduce the likelihood of overly small subgroups. Socioeconomic disadvantage was estimated using the Area Deprivation Index (ADI) [20]. A within-state ADI decile was assigned using each patient’s recorded home zip code. Since multiple ADI values could be associated with a single 5-digit zip code, when the ADI was mapped using a 5-digit zip code, the average of all ADI values for each 5-digit zip code was used. To reduce the likelihood of overly small subgroups, patients were grouped into ADI deciles of <  = 5 and > 5. Patients were excluded from those subgroups if an ADI could not be assigned (e.g., no matching zip code). Performance by level of rurality was assessed using Rural–Urban Continuum Codes (RUCC) [21], mapped using the patient’s home zip code and applying the suggested categorizations of codes 1–3 as metropolitan (“metro”) and codes 4–9 as non-metropolitan (“non-metro”). Patients were excluded from those subgroups if an RUCC code could not be assigned. See Fig. 1 for a graphical representation of inclusion/exclusion among groups.

### Statistical methods

Statistical comparisons were performed using R (version 4.2.0). Precision and recall were compared between the total population and the population stratified by demographic variables using two proportion z-tests with unequal sample sizes with a two-sided alternative hypothesis at 5% significance (alpha = 0.05). A Bonferroni correction for 24 tests for the pre-COVID dataset and 24 tests for the during-COVID dataset (the numbers of population and subset pairings) was used to adjust p-values for multiple comparisons within each performance metric (precision and recall). Post-hoc power analysis was done to determine the sample size required to detect a small Cohen’s h effect size (0.2) [22] for a two-proportion z-test with unequal sample sizes with a power of 0.80. Correlation coefficients were calculated using Pearson r correlations.

## Results

The datasets included 76,812 distinct inpatient visits, 47,750 prior to the COVID-19 pandemic and 29,062 during the pandemic.

AUC-ROC and AUC-PR for the pre-COVID dataset were 82% and 29% respectively, and 81% and 29% for the during-COVID dataset. No significant differences were found in precision or recall at either cutoff when comparing predictor performance on the full pre-COVID and during-COVID datasets (Table 2).

**Table 2:** Predictor validation pre- and during-COVID at selected cutoffs

**Metric**	**Cutoff**	**Pre-COVID Dataset**	**During-COVID Dataset**	**p-Value**
**Precision**	12.5%	25%	26%	0.230
**Recall**	12.5%	58%	59%	0.524
**Precision**	37.5%	44%	43%	0.840
**Recall**	37.5%	12%	11%	0.286

All comparisons had > 80% power

Model performance on each demographic subset of the pre-COVID dataset was compared to its overall performance on that dataset (Table 3). The only significant differences in precision or recall between a subgroup and the overall population were lower recall in the White non-Hispanic population at the 12.5% cutoff and lower recall in the non-metro population at both cutoffs. While a majority of the comparisons were adequately powered, a substantial minority were underpowered.

**Table 3:** Predictor performance for subgroups pre-COVID

		**12.5% Cutoff**				**37.5% Cutoff**			
Population	Preva-lence	Precision	Precision p-value	Recall	Recall p-value	Precision	Precision p-value	Recall	Recall p-value
All *n* = *47750*	0.08	0.25	–	0.58	–	0.44	–	0.12	–
Female *n* = *28265*	0.06	0.24	1	0.62	0.054	0.45	1*	0.13	1
Male *n* = *19485*	0.10	0.26	1	0.59	1	0.43	1*	0.12	1
White Non-Hispanic *n* = *40643*	0.09	0.25	1	0.53	**0.003**	0.45	1	0.11	1
Other Race/Ethnicity *n* = *6816*	0.05	0.22	0.90	0.66	0.121*	0.32	0.25*	0.12	1*
White Non-Hispanic Female *n* = *23523*	0.07	0.24	1	0.56	1	0.47	1*	0.11	1
White Non-Hispanic Male *n* = *17120*	0.11	0.26	1	0.55	0.40	0.44	1*	0.11	1
Other Race/Ethnicity Female *n* = *4574*	0.04	0.23	1	0.64	1*	0.32	1*	0.13	1*
Other Race/Ethnicity Male *n* = *2242*	0.08	0.22	1	0.59	1*	0.31	1*	0.09	1*
High ADI Rank *n* = *36571*	0.08	0.25	1	0.60	1	0.44	1	0.12	1
Low ADI Rank *n* = *10636*	0.07	0.25	1	0.60	1	0.42	1*	0.12	1
Metro *n* = *33663*	0.08	0.25	1	0.58	1	0.46	1	0.13	1
Non-metro *n* = *4325*	0.09	0.24	1	0.52	**0.021**	0.38	1*	0.08	**0.026**

Bold represent *p*-values < 0.05, therefore values that are statistically significantly different from the base comparison of “All”

*ADI* Area Deprivation Index – higher ADI values suggest greater levels of disadvantage

*asterisked items had Power < 80%; A Bonferroni correction for multiple comparisons was applied to the *p*-values; Prevalence is the fraction of patients in that group that died 5–90 days after the day of admission

For the during-COVID dataset (Table 4), compared to the overall population, the only significant differences among subgroups were lower precision in the Other Race/Ethnicity and the Other Race/Ethnicity female-only subgroups, but again, a substantial minority of comparisons (including all precision comparisons at the 37.5% cutoff) were underpowered.

**Table 4:** Predictor performance for subgroups during-COVID

		**12.5% Cutoff**				**37.5% Cutoff**			
Population	Preva-lence	Precision	Precision p-value	Recall	Recall p-value	Precision	Precision p-value	Recall	Recall p-value
All *n* = *29062*	0.09	0.26	–	0.59	–	0.43	–	0.11	–
Female *n* = *16948*	0.08	0.24	1	0.54	0.166	0.41	1*	0.10	1
Male *n* = *12114*	0.12	0.27	1	0.55	0.571	0.45	1*	0.10	1
White Non-Hispanic *n* = *22230*	0.10	0.27	1	0.58	1	0.45	1*	0.11	1
Other Race/Ethnicity *n* = *6585*	0.06	0.21	**0.004**	0.56	1*	0.33	1*	0.09	1*
White Non-Hispanic Female *n* = *12695*	0.08	0.26	1	0.61	1	0.44	1*	0.11	1
White Non-Hispanic Male *n* = *9544*	0.12	0.28	0.771	0.59	1	0.46	1*	0.11	1
Other Race/Ethnicity Female *n* = *4120*	0.05	0.18	**0.001**	0.53	1*	0.29	0.595*	0.09	1*
Other Race/Ethnicity Male *n* = *2473*	0.08	0.23	1	0.57	1*	0.40	1*	0.10	1*
High ADI Rank *n* = *21394*	0.10	0.26	1	0.55	0.423	0.44	1*	0.10	1
Low ADI Rank *n* = *7188*	0.08	0.25	1	0.58	1	0.41	1*	0.11	1
Metro *n* = *21249*	0.09	0.26	1	0.56	1	0.42	1*	0.10	1
Non-metro *n* = *2378*	0.11	0.27	1	0.60	1	0.46	1*	0.11	1

Bold represent *p*-values < 0.05, therefore values that are statistically significantly different from the base comparison of “All”

* ADI* Area Deprivation Index – higher ADI values suggest greater levels of disadvantage

*asterisked items had Power < 80%; A Bonferroni correction for multiple comparisons was applied to the *p*-values; Prevalence is the fraction of patients in that group that died 5–90 days after the day of admission

**Fig. 2:**
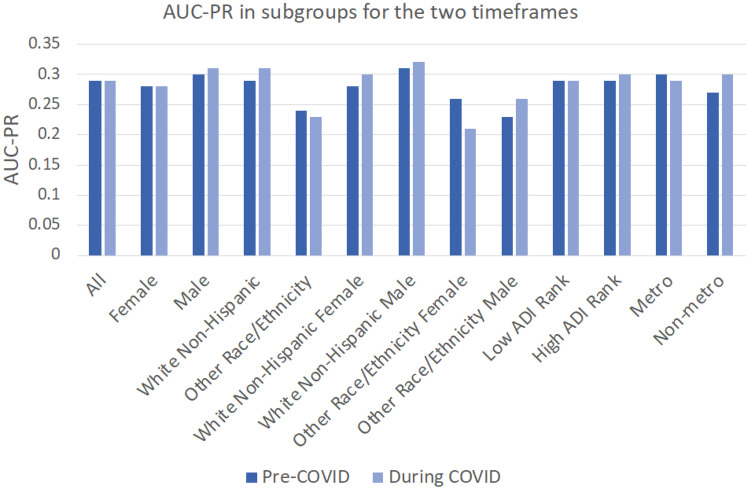
Area under the curve precision recall for subgroups in the pre-COVID and during-COVID periods

AUC-PR was also calculated for the subgroups (Fig. 2).

**Fig. 3:**
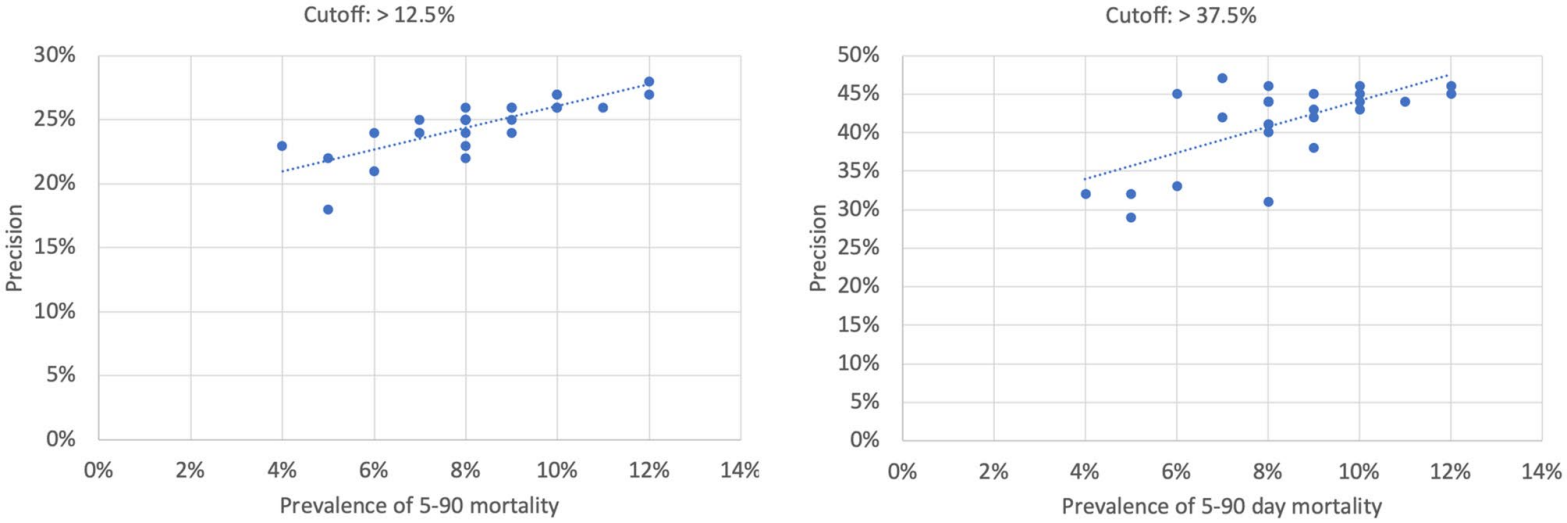
Prevalence of the outcome variable (5–90 mortality) vs. predictor precision in all studied populations and subgroups

Outcome variable prevalence affects predictor performance (particularly precision and AUC-PR) [23]. Therefore, we compared precision to 5-to-90-day mortality prevalence across all studied groups (Fig. 3). The Pearson r correlation coefficient between precision and prevalence was 0.79 (p < 0.001) at the 12.5% cutoff and 0.65 (p < 0.001) at the 37.5% cutoff.

## Discussion

ACP informs end-of-life care to respect patient preferences, ensure quality of life, and avoid costly, unnecessary, and unwanted interventions [2, 24]. Mortality prediction may help spur ACP conversations. Timely predictions may help strike a balance between sufficient clinical urgency and an adequate lead time to allow for these often time-consuming discussions [4, 25]. These predictions may be especially useful in mixed-rurality populations due to relatively reduced access to healthcare compared to urban populations.

This work was inspired by studies out of NYU Langone demonstrating the performance and impact of their 60-day mortality prediction model, originally intended to encourage appropriate patient referrals to supportive and palliative care [13]. Their model’s performance, with an AUC-PR of 28%, was also sufficient to achieve good rates of physician agreement with the alerts and greater use of ACPs [14]. Therefore, we sought similar performance for our model in our mixed-rurality population and to maintain that performance over time despite changing conditions. COVID-19 created significant systemic change in healthcare. Systemic change often causes performance degradation in machine learned models [16]. Our predictor demonstrated resistance to this concept drift, achieving an AUC-PR of 29% on both pre-COVID and during-COVID datasets.

NYU Langone selected a certainty cutoff providing 75% precision to identify likely-appropriate referrals to supportive and palliative care. The tradeoff for high precision was a recall of just 4.6% [13]. Since our intended use was solely to encourage ACP discussions, we evaluated two cutoffs designed to provide higher recall despite reduced precision. On the full pre-COVID dataset at a 12.5% certainty cutoff, our model achieved 58% recall and 25% precision; at a 37.5% cutoff the model achieved 12% recall and 44% precision. Model performance on the full during-COVID dataset did not significantly differ from that of the full pre-COVID dataset for any of those measures, demonstrating resistance to concept drift and performance degradation.

Previous work found racial differences in the relationship between physiologic and socioeconomic parameters and mortality prediction [26]. Many recommend accounting for potentially differing model performance among demographic groups [27–29]. The COVID-19 pandemic has disrupted healthcare, particularly affecting patients with low socioeconomic status [30, 31]. The timing and effectiveness of ACPs can be affected by socioeconomic circumstances, race, and geographic location [32, 33]. Given these considerations, we assessed model performance in different subgroups including rurality, level of socioeconomic disadvantage, gender, ethnicity, and race.

Significant performance differences were not seen for most comparisons, with notable exceptions and caveats. Recall was significantly lower than that of the overall pre-COVID population for White non-Hispanic patients and patients from non-metro areas. The reason for this is uncertain, but as discussed below, equity in precision may be more important than equity in recall for this use. Also, as the largest subpopulation, small relative performance differences for White non-Hispanic patients will more easily achieve statistical significance. During COVID, the Other Race/Ethnicity subgroup and its female-only subset had lower precision than the overall population (likely affected by the low 5-to-90-day mortality prevalence). Conclusions cannot be drawn and further research is warranted for a substantial minority of comparisons that were neither significantly different nor adequately powered. However, for the majority of comparisons, model performance was comparable to that of the overall population.

As expected, precision tended to be lower in subgroups having a lower 5-to-90-day mortality prevalence (Fig. 3). In the two instances for which precision was statistically significantly lower than the overall group, 5-to-90-day mortality prevalence was among the lowest of any subgroup. Since most precision comparisons were underpowered at the 37.5% cutoff, the 0.64 prevalence-to-precision correlation at that cutoff may be underestimated. This analysis shows that differences among subgroups in predicted risk at a particular cutoff are associated with actual differences in risk.

For subgroups having significant differences in model performance, the cutoffs for those subgroups could be adjusted to equalize performance. However, changing the cutoff typically improves either precision or recall while worsening the other, so one must select a metric to equalize. In our scenario, selecting cutoffs that equalize precision across subgroups would increase the likelihood that all who receive an alert will have a similar risk of near-term death. However, this means that subgroups with a lower prevalence of 5-to-90-day mortality will be less likely to receive an alert and therefore may less likely have an ACP. Instead, cutoffs could be selected to equalize recall across subgroups so that an equal fraction of patients who actually suffer a near-term death receive an alert. However, subgroups with a lower prevalence of 5-to-90-day mortality will be more likely to get an alert when they have a lower risk of death. This may lead to alert fatigue and/or mistrust of the predictor [18], and the magnitude of variation in cutoffs among demographic groups that would lead to predictor distrust in this context is not known. In addition, if clinician capacity for ACPs is limited, patients with lower 5-to-90-day mortality risk may get ACPs at the expense of those with greater urgency and need. Cutoffs could be selected to equalize the frequency of positive alerts across subgroups to equalize the predictor’s impact on ACPs across subgroups. As with equalizing on recall, however, this outcome may be lost if alerts on lower risk patients lead to alert fatigue and/or mistrust of the predictor. Also, those in greatest need of an ACP may be less likely to get one if clinician bandwidth to have ACPs is constrained. Other approaches may be taken, but all involve tradeoffs.

Existing literature suggests that equalizing the performance of a Boolean predictor among different subgroups is use-case dependent [17, 18]. For our use case, we suspect that equalizing precision across subgroups may best serve the clinical need by reducing the risk of alert fatigue and mistrust and prioritizing alerts to those with the greatest predicted need. However, since only a few statistically significant performance differences were seen among subgroups, and the statistical significance of those differences was inconsistent across the studied time periods, it may be wisest not to draw firm conclusions about whether or how to adjust cutoffs until the pandemic further stabilizes and the study can be repeated.

Our use of ADI to assess predictive model equity across levels of economic disadvantage along with the assessment of equity across different levels of rurality may be unique. A PubMed search on “ADI prediction equity” or “area deprivation index prediction equity” [34, 35] returned only one relevant result looking at the equity of a prediction model for various levels of ADI, and that study did not assess equity across levels of rurality [36].

### Limitations

Although assessments were designed to avoid use of data that will not be available at the time of prediction, complete avoidance cannot be guaranteed in this retrospective study. Other confounders related to the study’s retrospective nature may have affected results. This work was performed at one multi-hospital health system serving a predominantly White and Midwestern population, potentially limiting generalizability. Some demographic data may be inaccurate, affecting results. The ADI may not accurately represent the patient’s socioeconomic status, and our use of an average ADI for five-digit zip codes may not represent the patient’s census block ADI. Some demographies were aggregated to avoid small group sizes, and the predictor may perform differently across the aggregated demographies. Use of current code status as a proxy for status on admission may have affected results, but we believe patients are more likely to change from null or full code status to something else than the reverse. Our study was limited to model performance analysis, not its impact on clinical care. These limitations represent fruitful areas of future research.

## Conclusion

The predictor resisted concept drift and performance degradation from before to during the pandemic. Using precision for performance equitability assessment, although some precision comparisons (especially at the 37.5% cutoff) were underpowered and warrant further study, precision at the 12.5% cutoff was equitable across most demographies, regardless of the pandemic.

For time-constrained clinicians unable to have ACP discussions with every inpatient, this model may consistently and equitably help prioritize patients likely to benefit in the near-term from these crucial conversations.

### Supplementary Information

Below is the link to the electronic supplementary material.

Supplementary file1 (DOCX 21 KB)

## Data Availability

The datasets analyzed during the current study are not publicly available, since they were extracted from patients’ electronic health records. Data on patients are protected by medical confidentiality. The IRB approval includes the assurance that individual patient data will not be released. Data requests can be addressed to the corresponding author, who will evaluate the possibility of fulfilling the request considering institutional policies, regulatory requirements, and the patients’ privacy.

## References

[1] A. Brinkman-Stoppelenburg, J. A. C. Rietjens, and A. van der Heide, “The effects of advance care planning on end-of-life care: a systematic review,” *Palliat. Med.*, vol. 28, no. 8, Art. no. 8, Sep. 2014, 10.1177/0269216314526272. 10.1177/0269216314526272 24651708

[2] Martin RS, Hayes B, Gregorevic K, Lim WK (2016). The Effects of Advance Care Planning Interventions on Nursing Home Residents: A Systematic Review. J. Am. Med. Dir. Assoc..

[3] H. D. Lum, R. L. Sudore, and D. B. Bekelman, “Advance care planning in the elderly,” *Med. Clin. North Am.*, vol. 99, no. 2, Art. no. 2, Mar. 2015, 10.1016/j.mcna.2014.11.010. 10.1016/j.mcna.2014.11.010 25700590

[4] Dingfield LE, Kayser JB (2017). Integrating Advance Care Planning Into Practice. Chest.

[5] K. J. Johnston, H. Wen, and K. E. Joynt Maddox, “Lack Of Access To Specialists Associated With Mortality And Preventable Hospitalizations Of Rural Medicare Beneficiaries,” *Health Aff. (Millwood)*, vol. 38, no. 12, pp. 1993–2002, Dec. 2019, 10.1377/hlthaff.2019.00838. 10.1377/hlthaff.2019.00838 31794307

[6] B. Steffen-Bürgi, “[Ideas about a ‘good death’ in Palliative Care Nursing],” *Pflege*, vol. 22, no. 5, Art. no. 5, Oct. 2009, 10.1024/1012-5302.22.5.371. 10.1024/1012-5302.22.5.371 19780019

[7] H. Nelson-Brantley, C. Buller, C. Befort, E. Ellerbeck, A. Shifter, and S. Ellis, “Using Implementation Science to Further the Adoption and Implementation of Advance Care Planning in Rural Primary Care,” *J. Nurs. Scholarsh. Off. Publ. Sigma Theta Tau Int. Honor Soc. Nurs.*, vol. 52, no. 1, pp. 55–64, Jan. 2020, 10.1111/jnu.12513. 10.1111/jnu.12513 PMC6953624 31545557

[8] K. N. Yadav *et al.*, “Approximately One In Three US Adults Completes Any Type Of Advance Directive For End-Of-Life Care,” *Health Aff. Proj. Hope*, vol. 36, no. 7, Art. no. 7, Jul. 2017, 10.1377/hlthaff.2017.0175. 10.1377/hlthaff.2017.0175 28679811

[9] M. E. Charlson, P. Pompei, K. L. Ales, and C. R. MacKenzie, “A new method of classifying prognostic comorbidity in longitudinal studies: development and validation.,” *J. Chronic Dis.*, vol. 40, no. 5, Art. no. 5, 1987, 10.1016/0021-9681(87)90171-8. 10.1016/0021-9681(87)90171-8 3558716

[10] R. Venkataraman, V. Gopichandran, L. Ranganathan, S. Rajagopal, B. K. Abraham, and N. Ramakrishnan, “Mortality Prediction Using Acute Physiology and Chronic Health Evaluation II and Acute Physiology and Chronic Health Evaluation IV Scoring Systems: Is There a Difference?,” *Indian J. Crit. Care Med. Peer-Rev. Off. Publ. Indian Soc. Crit. Care Med.*, vol. 22, no. 5, pp. 332–335, May 2018, 10.4103/ijccm.IJCCM_422_17. 10.4103/ijccm.IJCCM_422_17 PMC5971641 29910542

[11] B. M. Sorger, B. Rosenfeld, H. Pessin, A. K. Timm, and J. Cimino, “Decision-making capacity in elderly, terminally ill patients with cancer.,” *Behav. Sci. Law*, vol. 25, no. 3, Art. no. 3, 2007, 10.1002/bsl.764. 10.1002/bsl.764 17506076

[12] S. Cohen *et al.*, “Communication of end-of-life decisions in European intensive care units.,” *Intensive Care Med.*, vol. 31, no. 9, Art. no. 9, Sep. 2005, 10.1007/s00134-005-2742-x. 10.1007/s00134-005-2742-x 16041519

[13] V. J. Major and Y. Aphinyanaphongs, “Development, implementation, and prospective validation of a model to predict 60-day end-of-life in hospitalized adults upon admission at three sites.,” *BMC Med. Inform. Decis. Mak.*, vol. 20, no. 1, p. 214, Sep. 2020, 10.1186/s12911-020-01235-6. 10.1186/s12911-020-01235-6 PMC7487547 32894128

[14] E. Wang *et al.*, “Supporting Acute Advance Care Planning with Precise, Timely Mortality Risk Predictions,” *NEJM Catal.*, vol. 2, no. 3, 2021, 10.1056/CAT.20.0655.

[15] W. Freeman, A. Weiss, and K. Heslin, “Overview of U.S. Hospital Stays in 2016: Variation by Geographic Region,” Agency for Healthcare Research and Quality, Rockville, MD, 246, Feb. 2018. [Online]. Available: www.hcup-us.ahrq.gov/nisoverview.jsp 30720972

[16] F. Bayram, B. S. Ahmed, and A. Kassler, “From concept drift to model degradation: An overview on performance-aware drift detectors,” *Knowl.-Based Syst.*, vol. 245, p. 108632, Jun. 2022, 10.1016/j.knosys.2022.108632.

[17] Paulus JK, Kent DM (2020). Predictably unequal: understanding and addressing concerns that algorithmic clinical prediction may increase health disparities. Npj Digit. Med..

[18] Rajkomar A, Hardt M, Howell MD, Corrado G, Chin MH (2018). Ensuring Fairness in Machine Learning to Advance Health Equity. Ann. Intern. Med..

[19] “ObituaryData.com,” Jul. 05, 2022. https://www.obituarydata.com/default.asp

[20] A. J. H. Kind and W. R. Buckingham, “Making Neighborhood-Disadvantage Metrics Accessible — The Neighborhood Atlas,” *N. Engl. J. Med.*, vol. 378, no. 26, pp. 2456–2458, Jun. 2018, 10.1056/NEJMp1802313. AND University of Wisconsin School of Medicine Public Health. Area Deprivation Index. Downloaded from https://www.neighborhoodatlas.medicine.wisc.edu/10.1056/NEJMp1802313 PMC6051533 29949490

[21] USDA Economic Research Service, “Rural-Urban Continuum Codes.” May 2013. Accessed: Jul. 21, 2022. [Online]. Available: https://www.ers.usda.gov/data-products/rural-urban-continuum-codes/documentation/

[22] J. Cohen, *Statistical power analysis for the behavioral sciences*, 2nd ed. Hillsdale, N.J.: L. Erlbaum Associates, 1988.

[23] S. Tenny and M. R. Hoffman, “Prevalence - StatPearls - NCBI Bookshelf,” *StatPerals*, 2020. https://www.ncbi.nlm.nih.gov/books/NBK430867/#:~:text=Prevalence%20thus%20impacts%20the%20positive,decreases%20while%20the%20NPV%20increases. (accessed Sep. 10, 2020).

[24] Detering KM, Hancock AD, Reade MC, Silvester W (2010). The impact of advance care planning on end of life care in elderly patients: randomised controlled trial. BMJ.

[25] Sudore RL (2017). Defining Advance Care Planning for Adults: A Consensus Definition From a Multidisciplinary Delphi Panel. J. Pain Symptom Manage..

[26] “RACIAL DIFFERENCES IN PREDICTING MORTALITY,” *The Gerontologist*, vol. 56, no. Suppl_3, pp. 506–507, Nov. 2016, 10.1093/geront/gnw162.2043.

[27] Gichoya JW (2022). AI recognition of patient race in medical imaging: a modelling study. Lancet Br. Ed..

[28] K. Palmer, “‘It’s not going to work’: Keeping race out of machine learning isn’t enough to avoid bias,” *STAT*.

[29] M. Tan *et al.*, “Including Social and Behavioral Determinants in Predictive Models: Trends, Challenges, and Opportunities.,” *JMIR Med. Inform.*, vol. 8, no. 9, p. e18084, Sep. 2020, 10.2196/18084. 10.2196/18084 PMC7509627 32897240

[30] A. N. Poudel *et al.*, “Impact of Covid-19 on health-related quality of life of patients: A structured review.,” *PloS One*, vol. 16(10): e0259164, 2021, 10.1371/journal.pone.0259164. 10.1371/journal.pone.0259164 PMC8553121 34710173

[31] Gold JAW (2022). Dispensing of Oral Antiviral Drugs for Treatment of COVID-19 by Zip Code-Level Social Vulnerability - United States, December 23, 2021-May 21, 2022. MMWR Morb. Mortal. Wkly. Rep..

[32] Tripken JL, Elrod C, Bills S (2018). Factors Influencing Advance Care Planning Among Older Adults in Two Socioeconomically Diverse Living Communities. Am. J. Hosp. Palliat. Care.

[33] Khosla N, Curl AL, Washington KT (2016). Trends in Engagement in Advance Care Planning Behaviors and the Role of Socioeconomic Status. Am. J. Hosp. Palliat. Care.

[34] “prediction equity area deprivation index - Search Results - PubMed,” *PubMed*. https://pubmed.ncbi.nlm.nih.gov/?term=prediction%20equity%20area%20deprivation%20index (accessed Sep. 13, 2022).

[35] “adi prediction equity - Search Results - PubMed,” *PubMed*. https://pubmed.ncbi.nlm.nih.gov/?term=prediction%20equity%20adi (accessed Sep. 13, 2022).

[36] Weissman GE, Teeple S, Eneanya ND, Hubbard RA, Kangovi S (2021). Effects of neighborhood-level data on performance and algorithmic equity of a model that predicts 30-day heart failure readmissions at an urban academic medical center. J. Card. Fail..

