# Supplementary material for: Can a 5-to-90-day Mortality Predictor Perform Consistently Across Time and Equitably Across Populations?
Source: J Med Syst. 2023 Jul 3;47(1):67. doi: 10.1007/s10916-023-01962-z (PMC10317873; doi:10.1007/s10916-023-01962-z)
Supplement: Supplementary file 1 — Supplementary file1 (DOCX 21 KB) [file 10916_2023_1962_MOESM1_ESM.docx]

Supplement

**Title of Paper:** Can a 5-to-90-day Mortality Predictor Perform Consistently Across Time and Equitably Across Populations?

**Authors:** Jonathan Handler^1, 6^, Olivia J. Lee^2^, Sheena Chatrath^2^, Jeremy McGarvey^3^, Tyler Fitch^4^, Divya Jose^5^, John Vozenilek^2, 7, 8^

^1^Clinical Intelligence and Advanced Data Lab, OSF Healthcare System, Peoria, IL, USA

^2^University of Illinois College of Medicine at Peoria, Peoria, IL, USA

^3^Ministry Healthcare Analytics, OSF HealthCare System, Peoria, IL, USA

^4^Internal Medicine and Pediatrics, OSF Healthcare System, Peoria, IL USA

^5^Business Intelligence Consulting, Indus Group, Wheeling, IL, USA

^6^Department of Emergency Medicine, Northwestern University Feinberg School of Medicine, Chicago, IL, USA

^7^OSF Innovation, OSF Healthcare System, Peoria, IL, USA

^8^University of Illinois College of Engineering, Urbana Champaign, IL USA

**Corresponding author**: Jonathan Handler, 1306 N Berkeley Ave, Peoria, IL 61603, jonathan.a.handler@osfhealthcare.org, (309) 677-0810

**Acknowledgements**

The authors would like to thank Safura Sultana for her support in obtaining IRB approval, project management administration, and manuscript support.

# Model Development in Greater Depth

As part of a quality improvement effort prior to this study, the Boolean mortality predictor model was built using Python[1] with the scikit-learn[2] and CatBoost[3]–[5] libraries. CatBoost, a tree-based model, was selected because it is known for its strong performance,[6] it is considered to have very good automated support for handling categorical variables (hence the first part of its name of CatBoost), and it has mechanisms to automatically handle missing data. Specifically, its default behavior to handle missing data is to handle missing (null) categorical data as its own category (empty string),[3], [7] and to assign a minimum value to any numeric data (lower than any other value in the dataset). For numeric data, CatBoost documentation says it is “guaranteed that a split that separates missing values from all other values is considered when selecting trees.”[8] Based on this, we infer that the model can treat missing data differently than other data as needed.

The model was derived on a dataset randomly split by entity (patient) using scikit-learn’s GroupShuffleSplit algorithm [[18]](https://www.zotero.org/google-docs/?oNPgZR) into training, validation, and holdout datasets (63% used for training, 7% for validation, and 30% for holdout testing). This ensured that all visits from a patient were entirely in one of those datasets (training, validation, or holdout). We believe this reduced the likelihood of reporting results that would be inapplicable on an entirely new set of patients.

Input features were chosen by a clinical domain expert (physician informaticist) as likely to be predictive of mortality, and as having high face-validity for users of the model. In other words, features were chosen that other clinicians would likely accept and understand as indicating a greater likelihood of mortality. For example, the lab tests selected as inputs were chosen because they may indicate poor functioning or failure of a vital organ or body system. Having input features with high face validity was desirable to increase the likelihood of acceptance of the model and to facilitate interpretability and plausibility on individual clinician review of the model’s predictions in practice. Extensive feature engineering of the input features was done to enhance model performance. The feature engineering created new input features by combining input features, trending features (change in a feature between one time period and another), and applying aggregate functions to features (e.g., minimum value over a time period, maximum value over a time period, etc.). All features (including the engineered features) are generated from a single SQL database query, albeit one composed of multiple common table expressions (akin to subqueries).

The final model’s inputs were selected automatically using a forward feature selection algorithm. In this approach, input features were added sequentially in order of importance to the model (as determined by an original training run using all inputs), and each input was retained only if the performance of the algorithm (AUC-PR) on the validation set with the input included was higher than the performance without it.

# Supplement Bibiliography

[1] “Python.” https://www.python.org/about/

[2] “scikit-learn.” https://scikit-learn.org/stable/

[3] L. Prokhorenkova, G. Gusev, A. Vorobev, A. V. Dorogush, and A. Gulin, “CatBoost: unbiased boosting with categorical features.” arXiv, Jan. 20, 2019. Accessed: Mar. 14, 2023. [Online]. Available: http://arxiv.org/abs/1706.09516

[4] A. V. Dorogush, V. Ershov, and A. Gulin, “CatBoost: gradient boosting with categorical features support,” *ArXiv*, vol. abs/1810.11363, 2018.

[5] “CatBoost - state-of-the-art open-source gradient boosting library with categorical features support.” https://catboost.ai (accessed Apr. 25, 2022).

[6] “CatBoost Classifier in Python.” https://kaggle.com/code/prashant111/catboost-classifier-in-python (accessed Mar. 14, 2023).

[7] “How does catboost handle missing categorical values during categorical encoding and predicting? · Issue #1245 · catboost/catboost,” *GitHub*. https://github.com/catboost/catboost/issues/1245 (accessed Mar. 14, 2023).

[8] “Missing values processing.” https://catboost.ai/en/docs/concepts/algorithm-missing-values-processing (accessed Mar. 14, 2023).
